# Cervical vertebral and spinal cord injuries in rollover occupants

**DOI:** 10.1186/s40621-024-00506-4

**Published:** 2024-07-03

**Authors:** Loay Al-Salehi, Shannon G. Kroeker, Jason R. Kerrigan, Peter A. Cripton, Matthew B. Panzer, Gunter P. Siegmund

**Affiliations:** 1 Orthopedic and Injury Biomechanics Group, School of Biomedical Engineering, Departments of Orthopaedics and Mechanical Engineering, University of British Columbia, Vancouver, BC Canada; 2 MEA Forensic Engineers & Scientists, 23281 Vista Grande Drive, Laguna Hills, CA 92653 USA; 3 Center for Applied Biomechanics, School of Engineering and Applied Science, University of Virginia, Charlottesville, VA USA; 4 School of Kinesiology, University of British Columbia, Vancouver, BC Canada

## Abstract

**Background:**

Rollover crashes continue to be a substantial public health issue in North America. Previous research has shown that the cervical spine is the most injured spine segment in rollovers, but much of the past research has focused on risk factors rather than the actual cervical spine injuries. We sought to examine how different types of cervical spine injuries (vertebral and/or cord injury) vary with different occupant-related factors in rollovers and to compare these with non-rollovers.

**Methods:**

We obtained crash and injury information from the National Automotive Sampling System–Crashworthiness Data System (NASS-CDS) for 2005–2015 and Crash Investigation Sampling System (CISS) for 2017–2022. Based on weighted data, we calculated relative risks to assess how occupant sex, seat belt use, ejection status, and fatal outcome relate to the rate of different cervical spine injuries in rollovers and non-rollovers.

**Results:**

In NASS-CDS occupants with cervical spine injuries (*N* = 111,040 weighted cases), about 91.5% experienced at least one vertebral injury whereas only 11.3% experienced a spinal cord injury (most of which had a concomitant vertebral fracture). All types of cervical spine injuries we examined were 3.4–5.2 times more likely to occur in rollovers compared to non-rollovers. These relative risks were similar for both sexes, belted and unbelted, non-ejected, and non-fatal occupants. The number of weighted CISS occupants with cervical spine injuries (*N* = 42,003) was smaller than in the NASS analysis, but cervical spine injuries remained 6.25 to 6.36 times more likely in rollovers compared to non-rollovers despite a more modern vehicle fleet.

**Conclusions:**

These findings underscore the continued need for rollover-specific safety countermeasures, especially those focused on cervical spine injury prevention, and elucidate the frequency, severity and other characteristics of the specific vertebral and spinal cord injuries being sustained in rollovers. Our findings suggest that countermeasures focused on preventing cervical vertebral fractures will also effectively prevent most cervical spinal cord injuries.

**Supplementary Information:**

The online version contains supplementary material available at 10.1186/s40621-024-00506-4.

## Background

Motor vehicle rollover crashes remain a public health concern in North America and elsewhere in the world despite improvements in vehicle stability and passive safety. In the United States, rollovers account for only 2% of all motor-vehicle crashes yet cause almost a third of all vehicle-related fatalities (National Center for Statistics and Analysis 2020). Rollovers also cause many non-fatal cervical spine fractures, dislocations, and spinal cord injuries, some of which are catastrophically life-altering for the injured individuals and include tetraplegia and ventilator dependence. The mechanism of many of these injuries has been shown in rollover tests with anthropomorphic tests devices (ATDs) where the ATDs sustain headfirst impacts with the vehicle’s roof, during roof-to-ground impacts while the rolling vehicle is inverted (Bahling et al. 1990; Cooper et al. 1997; Moffatt et al. 2003; Raddin et al. 2009). These injuries to the cervical spine create sizeable societal burdens on national economies and healthcare systems (Berkowitz 1998; Burns et al. 2010; Digges 2002; Ma et al. 2014; National Spinal Cord Injury Statistical Center 2020). Although the magnitude of the societal and health care problems caused by cervical spine injuries in automotive rollovers is understood, there is a paucity of information on the rates and characteristics of different cervical spine injuries in rollover crashes. In order to prevent these injuries, researchers and manufacturers need to understand the injury mechanisms responsible. This information is of primary importance to enable prevention of future cervical spine injuries in rollover crashes.

Considerable epidemiological data exist describing injury characteristics in rollover occupants (Bose et al. 2011; Fakharian et al. 2017; Foster et al. 2012; Funk et al. 2012; Ivarsson et al. 2015; Mandell et al. 2010; McMurry et al. 2016; Parenteau and Viano 2014; Ridella and Eigen 2008; Stein et al. 2011). The occurrence and severity of various injuries in rollovers have been associated with seating position (Funk et al. 2012; Jehle et al. 2007; Viano et al. 2007), occupant age (Bilston et al. 2011; McMurry et al. 2016; Stein et al. 2011), ejection status (Funk et al. 2012; Gloeckner et al. 2006), and seatbelt use (Funk et al. 2012; Moore, 2005; Parker, 2007; Viano et al. 2007), but none of these studies focused specifically on cervical spine injuries in rollover crashes. Parenteau and Viano (2014) examined injury severity (using the Abbreviated Injury Scale, AIS) along the entire spine and found that occupants in rollovers had the highest rate of AIS4 + cord injuries and AIS3 fracture-dislocations compared to other collision types. They also noted that 69.5% of all AIS4+ spinal cord injuries in rollovers were to the cervical spine, but did not explore further the types and characteristics of cervical spine injuries in rollover crashes. Stein et al. (2011) found that vertebral column fractures and spinal cord injuries in the cervical spine were 5.3 to 6.5 times more likely in rollover crashes than in frontal crashes. These authors reported no differences in the crash characteristics (e.g., crash type, seat belt use, etc.) or occupant characteristics (e.g., sex, age, etc.) between occupants with cervical spine fractures versus spinal cord injuries, but these findings were based on only 57 occupants who experienced rollovers amongst the 407 occupants with cervical spine injuries in the CIREN^1^ database at the time. To our knowledge, an analysis of population-weighted data that distinguishes between spinal cord injuries with and without fractures, as well as vertebral fractures that occur with and without cord injuries, has not been reported. Despite Stein et al.’s and Viano et al.’s detailed analyses, the relative frequency and relative risk of different types of cervical spine injuries in rollover and non-rollover crashes remain unexamined.

To address this gap in the literature, the goal of the current study is to examine how the different types and patterns of cervical spine injuries (e.g., vertebral fracture, spinal cord injury, etc.) vary with different occupant-related factors in rollover and (for comparison) non-rollover crashes. More specifically, we sought to determine: (i) the rate of cervical spine injuries in rollover and non-rollover crashes, (ii) how occupant sex, seat belt use, ejection status, and fatal outcome relate to the rates of cervical spine injuries in rollover and non-rollover crashes, and (iii) which types of vertebral injuries are associated with spinal cord injuries in rollover and non-rollover crashes.

## Methods

We extracted data from two multi-site US databases: the National Automotive Sampling System – Crashworthiness Data System (NASS-CDS) and its more recent replacement the Crash Investigation Sampling System (CISS) to examine both older and newer vehicles. Because of differences in the databases (Zhang et al. 2019) and our desire to compare the relative risks between an older and newer fleet, we performed separate analyses for each dataset.

### NASS-CDS analysis

The NASS-CDS database compiled by the National Highway Traffic Safety Administration (NHTSA) is a probability sample of police-reported tow-away crashes and was used to query real-world crash and injury data for our analysis. Annually, detailed crash and injury data from about 5000 crashes were studied in 24 geographically distributed “Primary Sampling Units” (PSUs) across the USA and stored in the publicly available NASS-CDS database. The NASS-CDS provides the raw counts as well as ratio inflation factors that weight the raw data to represent estimates of all police-reported, motor-vehicle, tow-away crashes occurring in the USA in a given year up to 2015 (Radja 2016; Zhang et al. 2019). NASS-CDS data consist of multiple sub-records, which include the accident (ACCIDENT), general vehicle (GV), occupant assessment (OA), occupant injury (OI), vehicle exterior (VE), vehicle interior (VI), and accident event (EVENT) records. To avoid the loss of data rows, these seven sub-records were synthesized in SAS 9.4 (SAS Institute, Cary, NC) for each year using the *PROC SQL* function in the abovementioned order. The complete dataset was created by merging synthesized sub-records for the years 2005–2015 in SAS Enterprise 7.1 (SAS Institute, Cary, NC). For clarity, NASS-CDS variable names are capitalized in the following text.

To select the data used for our NASS-CDS analysis, we first filtered the merged data for criteria related to vehicle and occupant details. Only passenger cars and light trucks (BODYTYPE ≤ 49) with model years of 1985 or newer (MODELYR ≥ 1985) were included in our analysis. Excluding pre-1985 vehicles for crashes only eliminated < 1% of vehicles (728 of 84,659 vehicles) and about 1.5% of occupants (1811 of 117,816 occupants). Occupants over 8 years old (AGE > 8) and taller than 145 cm (HEIGHT > 145) were included. These criteria included 5th percent females (McDowell, 2008), but excluded very short individuals, and were consistent with age and height restrictions for booster seat use (i.e. most occupants shorter than those included would be mandated to use child booster seats) legislated by most states with NASS-CDS PSUs (“State Laws,” n.d.; United States Government Accountability Office 2015). Occupants were also excluded if they were using a child restraint of any kind (CHTYPE > 0).

The dependent variables for all four of our study goals were the type and combination of cervical spine injuries, which were classified using the 1998 Abbreviated Injury Scale code (AIS98) in the NASS-CDS database (Association for the Advancement of Automotive Medicine 2008). All cervical spine injuries (REGION90 = 6, STRUSPEC = 2) were identified and first categorized into three main groups (Table 1). The first group consisted of occupants with vertebral injuries only (abbreviated VI in Table 1) and was defined by any number of fractures or dislocations to the cervical vertebrae (STRUTYPE = 5 and INJLEVEL = 04, 06 to 34 inclusive) without any spinal cord involvement (STRUTYPE ≠ 4). The second group consisted of occupants with spinal cord injuries (abbreviated CI in Table 1) and was defined as any number of spinal cord injuries with or without associated fractures or dislocations (STRUTYPE = 4, INJLEVEL = 00 to 76 inclusive) and without any fractures or dislocations unrelated to the spinal cord injury (STRUTYPE ≠ 5). The third group consisted of occupants who had both a spinal cord injury (STRUTYPE = 4, INJLEVEL = 00 to 76 inclusive) and a separate, distinct vertebral fracture or dislocation (STRUTYPE = 5 and INJLEVEL = 04 to 34 inclusive). This third group of vertebral and cord injuries was denoted by the abbreviation VCI in Table 1. Each occupant was included in only one of these three groups. In addition to these three main groups, we also considered combinations of the groups: all occupants with vertebral injuries (All-VI = VI + VCI), all occupants with spinal cord injuries (All-CI = CI + VCI), and all occupants with vertebral and/or spinal cord injuries (ALLINJ = VI + CI + VCI). Occupants with only ligamentous injuries or strains (STRUTYPE = 4 and INJLEVEL = 84 or 78), intervertebral disc injuries (STRUTYPE = 5 and INJLEVEL = 00, 02, 03 or 99), and nerve root injuries (STRUTYPE = 3), whether isolated or combined, were excluded from all groups to isolate only spinal cord and bony vertebral injuries. Based on these definitions, the AIS values for all VIs varied from AIS2 to AIS3 and all CIs varied from AIS3 to AIS6. All included injuries require physical examination or imaging to be diagnosed.

**Table 1:** Summary of the three main groups of cervical spine injuries. Subscripts i and j denote different AIS-coded injuries within the same individual’s cervical spine

Group	Description of cervical spine injury	Conditions
VI	Vertebral injury only - One or more fractures or dislocations with no spinal cord injury	(STRUTYPE_i_=5) & (INJLEVEL_i_=04 to 34) & (STRUTYPE_j_≠4)
CI	Spinal cord injury - One or more spinal cord injuries with or without associated fracture or dislocation; no other vertebral injury without spinal cord injury	(STRUTYPE_i_=4) & (INJLEVEL_i_=00 to 76) & (STRUTYPE_j_≠5)
VCI	One or more spinal cord injuries with or without fracture or dislocation PLUS one or more fractures or dislocations with no spinal cord injury	(STRUTYPE_i_=5) & (INJLEVEL_i_=04 to 34) & (STRUTYPE_j_=4) & (INJLEVEL_j_=00 to 76)

To achieve our third goal, i.e., how different types of vertebral injuries combine with spinal cord injuries in rollover and non-rollover crashes, we further categorized the occupants with spinal cord injuries (the All-CI group) based on their associated vertebral injuries into the following five subgroups:

no fractures or dislocations

(INJLEVEL=2,12,22,30,44,62,70), fractures (INJLEVEL=4,14,24,32,46,64,72), dislocations

(INJLEVEL=6,16,26,34,48,66,74), combined fracture-dislocations

(INJLEVEL=8,18,28,36,50,68,76) and not further specified (NFS)

(INJLEVEL=0,1,10,20,21,29,40,42,60,61,69).

The primary independent variables for our analysis consisted of the collision type (rollover versus non-rollover), seat belt use, ejection status, and fatality status. Vehicles were categorized according to whether they experienced a rollover crash (ROLLOVER = 1 to 12 quarter turns) or non-rollover crash (ROLLOVER = 0 quarter turns). We discarded rollovers of more than 3 revolutions (ROLLOVER > 12 quarter turns), end-over-end rollovers (ROLLOVER = 98) due to their rare occurrence (< 1% of NASS and CISS cases) and substantially different kinematics, and any crashes coded as unknown (ROLLOVER = blank; 65 vehicles, 99 occupants). Occupants were categorized as belted if they used a manual lap and shoulder belt (MANUSE = 4) or an automatic belt system (ABELTUSE = 1), and categorized as unbelted otherwise. We discarded occupants missing both MANUSE and ABELTUSE data. Occupants were classified as ejected (EJECTION = 1) or not ejected (EJECTION = 0), and were discarded otherwise. Occupants were only categorized as being fatally injured when TREATMNT = 1. For the latter three independent variables, i.e., seat belt use, ejection status or fatality status, we excluded occupants on an analysis-by-analysis basis. For example, an occupant with missing seat belt data was only excluded from the seatbelt analysis.

We also conducted a separate sub-analysis wherein we attempted to select occupants who likely sustained their cervical spine injury inside the vehicle during the rollover portion of the crash. This has been done previously (Bose et al. 2011; Funk et al. 2012; McMurry et al. 2016). For this sub-analysis, we only included occupants in rollovers who met all of the following additional criteria: (i) the first or second most severe crash event was the rollover (OBJCONT1 = 31 or OBJCONT2 = 31), (ii) the rollover was not an end-over-end rollover (ROLINDIR ≠ 8 or blank, or OBJCONT ≠ 32), (iii) at least half a roll occurred (ROLLOVER ≥ 2), (iv) a collision with another vehicle did not initiate the rollover (ROLINTYP ≠ 7), and (v) the occupant was not completely ejected (EJECTION ≠ 1). Occupants who were partially ejected (EJECTION = 2) were only included if their cervical spine injury was caused by sources within the vehicle (INJSOU < 451 or INJSOU = 570, 572, 575, 576, or 602).

### CISS analysis

We repeated the foregoing analyses using the CISS database to evaluate the impact of advancements in the field of passive safety over the last decade and derive conclusions more relevant to the current vehicle fleet. CISS is NHTSA’s newer database of nationally collected surveys of police-reported vehicle crashes (Radja et al. 2023; Zhang et al. 2019) and, compared to NASS-CDS, prioritizes the selection of newer vehicles (≤ 4 years old) that are more likely to be equipped with advanced crashworthiness and crash-avoidance technologies (Mynatt and Brophy 2017). We combined CISS data files from 2017 to 2022 and excluded data from the 2016 pilot year. Similar to our NASS-CDS analysis, we filtered the merged data for vehicle type (BODYTYPE ≤ 49), occupant age (AGE > 8), occupant height (HEIGHT > 145), and excluded child restraint use (CHTYPE > 0). Only vehicle model years 2010 and newer (MODELYR ≥ 2010) were included, as these vehicles will likely include advancements in rollover safety due to introduced safety legislation, e.g. relating to roof strength (FMVSS No. 216a, 2009) and ejection mitigation (FMVSS No. 226, 2011).

Since the CISS dataset was smaller than the NASS-CDS dataset and some of the injury categories contained only a few raw cases (e.g., there were only 5 raw VCI cases), we focused our CISS analysis on the All-VI, All-CI, and All-Injuries groups. Occupant injuries in CISS are described using the 2015 AIS code (AIS15, The Association for Advancement of Automotive Medicine 2016), which required slightly different definitions for our injury groups. For our CISS analysis, vertebral injury (VI) was defined by any number of fractures or dislocations to the cervical vertebrae (STRUTYPE = 5 and INJLEVEL = 04, 06 to 40 inclusive) without spinal cord involvement (STRUTYPE ≠ 1) and cord injury (CI) was defined as any number of spinal cord injuries with or without associated fractures or dislocations (STRUTYPE = 1, INJLEVEL = 00 to 36 inclusive) and without any fractures or dislocations unrelated to the spinal cord injury (STRUTYPE ≠ 5). Both the All-VI and All-CI groups contained the 5 occupants who had both a VI and CI. Duplicate occupants were removed before the analyses.

We analyzed the same independent variables we used in the NASS-CDS analysis, but adjusted some definitions to accommodate CISS’s revised variable structure: both seat belt use (BELTUSE = 4) and fatality (MORTALITY = 1) were redefined. Since the analyses only encompassed vehicles manufactured after 2010, the infrequent instances of a distinct lap-(BELTUSE = 3) and shoulder belt (BELTUSE = 2) were negligible in terms of statistical strength and allowed us to control for one seatbelt type. For our analysis of how different types of vertebral injuries combined with spinal cord injuries (All-CI group) in both rollover and non-rollover crashes, our definitions underwent the following AIS15-related changes: no fractures or dislocations (INJLEVEL = 2,12,22,30), fractures (INJLEVEL = 4,14,24,32), dislocations (INJLEVEL = 6,16,26,34), combined fracture-dislocations (INJLEVEL = 8,18,28,36) and not further specified (NFS) (INJLEVEL = 0,1,10,20,21,29). To filter for occupants who likely sustained their cervical spine injury inside the vehicle during the rollover portion of the crash, we also performed a sub-analysis where we only included occupants who, similar to the equivalent NASS-CDS analysis, met all of the following additional criteria: (i) the first or second most severe crash event was the rollover (OBJCONT = 31 and DVRANK = 1 or 2), (ii) the rollover was not an end-over-end rollover (ROLLTYPE = 1), (iii) at least half a roll occurred (ROLLTURN ≥ 2), (iv) a collision with another vehicle did not initiate the rollover (1 ≤ ROLLINITYP ≤ 6), and (v) the occupant was not completely ejected (EJECTTYPE ≠ 1). Occupants who were partially ejected (EJECTTYPE = 2) were only included if their cervical spine injury was caused by sources within the vehicle (IPC1 < 1100 or 1501 ≤ IPC1 ≤ 1698).

### Statistical analysis

All descriptive statistical analyses were performed in SAS 9.4 (SAS Institute, Cary, NC) using the SAS *SURVEYFREQ* procedures for analyzing complex sample surveys. We used the default variance estimation method, i.e., a Taylor series approximation. We calculated population totals (weighted data), as well as population proportions and relative risks for 2 × 2 frequency tables from the weighted data for each injury category. We also calculated the 95th percentile confidence intervals for population totals, proportions, and relative risks. In NASS-CDS, from 2002 to 2007, three extra so-called alliance PSUs were deleted and the weighting factors for the remaining PSUs were adjusted. Relative risks (RR, Eq. 1) and their confidence intervals were calculated in SAS, and used to compare rollover and non-rollover risks for each cervical spine injury category. Relative risks were considered significant if the 95th -percentile confidence interval (CI) did not include 1. Relative risks between independent subgroups (e.g., males and females), and relative risks in NASS-CDS and CISS were compared using a test of interaction at a significance level of α ≤ 0.05 (Altman and Bland 2003).

1 $$RR=\frac{\left(\frac{{N}_{rollover}^{injured}}{{N}_{rollover}^{all}}\right)}{\left(\frac{{N}_{nonrollover}^{injured}}{{N}_{nonrollover}^{all}}\right)}$$

## Results

### NASS-CDS analysis

Of all occupants included in our NASS-CDS analysis, 7.3% (1.685 M/23.204 M) or 153,169/year experienced a rollover crash and about 0.5% of all occupants (0.111 M/23.204 M) or 10,095/year experienced a cervical spine injury (Table 2). Amongst all occupants with cervical spine injuries, about 91.5% (101.7k/111.0k) experienced at least one vertebral injury, whereas only 11.3% (12.6k/111.0k) experienced a spinal cord injury. Both of these proportions were similar for rollover occupants (93.7% and 9.1%, respectively) and non-rollover occupants (90.7% and 12.2%, respectively).

**Table 2:** Summary of the (a) raw counts and (b) weighted data for the three primary injury categories (top three rows of each table), the three combined injury categories (middle three rows), and all exposed occupants (bottom row). Also shown are the 95th percentile confidence intervals (CI) for the weighted data

a) Raw counts						
Injury Category	Rollover		Non-Rollover		Total	
VI - Vertebral Injuries only	368		800		1168	
CI – Cord injuries	39		153		192	
VCI – Separate VI and CI	16		24		40	
All-VI (VI + VCI)	384		824		1208	
All-CI (CI + VCI)	55		177		232	
All-Injuries (VI + CI + VCI)	423		977		1400	
All Exposed Occupants	5,776		45,884		51,660	
b) Weighted data						
Injury Category	Rollover	(95th CI)	Non-Rollover	(95th CI)	Total	(95th CI)
VI - Vertebral Injuries only	28,377	(14,559 − 42,195)	70,062	(48,975 − 91,148)	98,439	(68,069–128,808)
CI – Cord injuries	1,975	(661–3,288)	7,412	(3,435 − 11,389)	9,387	(4,296 − 14,477)
VCI – Separate VI and CI	885	(0–1,888)	2,330	(0–5,180)	3,215	(262–6,167)
All-VI (VI + VCI)	29,262	(15,080 − 43,443)	72,392	(51,334 − 93,450)	101,653	(70,927 − 132,380)
All-CI (CI + VCI)	2,859	(871–4,848)	9,742	(4,997 − 14,487)	12,601	(6,909 − 18,293)
All-Injuries (VI + CI + VCI)	31,236	(15,839 − 46,633)	79,804	(57,078–102,530)	111,040	(76,466 − 145,614)
All Exposed Occupants	1,684,859	(1,165,772–2,203,947)	21,519,968	(15,312,915 − 27,727,022)	23,204,828	(16,533,414 − 29,876,242)

Although most cervical spine injuries occurred in non-rollover crashes (Table 2b), proportionally more cervical spine injuries occurred in rollover crashes than in non-rollover crashes. Despite rollovers accounting for only 7.2–7.4% of occupant exposures (Table 3a), between 21.0% (14.4–27.6%) of cervical cord injuries (CI) and 28.8% (20.1–37.4%) (mean and 95th percentile confidence interval) of all cervical vertebral injuries (All-VI) occurred in rollover crashes (Table 4a). This general pattern was observed separately in the female and male subpopulations, the belted and unbelted subpopulations, and in the non-ejected and non-fatal subpopulations (compare the rollover percentages in Tables 3a and 3b). It was not observed in the ejected and fatal subpopulations.

Compared to all occupants involved in non-rollover crashes, the relative risks for the cervical spine injuries in a rollover ranged from 3.40 (2.28–5.09) for occupants with a cord injury (CI group) to 5.17 (3.43–7.81) for occupants with only a cervical vertebral injury (VI group) (top row of relative risks in Table 4a). These relative risks were significantly greater than 1 for all injury categories, except for occupants with separate vertebral and spinal cord injuries (VCI group), which contained only 40 raw cases (3215 weighted cases) and thus had a large confidence interval (Table 2).

A similar pattern of increased risk for cervical spine injuries in rollover crashes was present in most of the subpopulations we examined (Table 4a). Across the female and male subpopulations, the belted and unbelted subpopulations, and both the non-ejected and non-fatal subpopulations the relative risks varied from 3.03 (1.75–5.25) to 11.31 (2.13–59.90) (Table 4a). A different pattern was observed, however, in the ejected and fatal subpopulations. In the ejected subpopulation, there was no difference in risk for the injury categories dominated by vertebral injuries (VI, All-VI and All Injuries groups), whereas the cord-related injury categories (CI, VCI and All-CI groups) had a lower risk of injury in rollover crashes compared to non-rollover crashes. The pattern within the fatal subpopulation was less clear, with the VI, VCI and All-VI groups showing no difference in risk, and the CI, All-CI and All Injuries groups showing a lower risk of injury in rollover crashes compared to non-rollover crashes.

When we performed a sub-analysis using only non-ejected occupants whose injuries likely occurred inside the rolling vehicle, the proportions of injuries associated with rollovers diminished to between 8.0% (0.1–15.8%) for the CI group and 16.7% (10.3–23.1%) for the VI group, and only the VI, All-VI and All-Injuries groups had relative risks significantly greater than 1 (Table 4b). The other three injury categories had relative risks that were not significantly different from 1.

**Table 3:** Weighted counts and proportions (%) for (a) all exposed occupants (injured and uninjured) and (b) all injured occupants (occupants with cervical spine injuries) separated by their exposure to a rollover or non-rollover crash. Separate analyses are tabulated for cases where sex, seatbelt use, ejection status and fatality information was known. Abbreviations: R = rollover, NR = non-rollover, N = weighted counts, Nall = total weighted counts for each condition. The percentages for the rollover (R/N) and non-rollover (NR/N) conditions for each row sum to 100%, whereas the total column percentages (N/Nall) for each group sum to 100%

a) All Exposed Occupants						
	Rollover		Non-Rollover		Total	
Group	R	R/N	NR	NR/N	N	N/Nall
Sex						
Female	641,371	5.6%	10,769,883	94.4%	11,411,253	49.2%
Male	1,043,477	8.9%	10,746,176	91.1%	11,789,653	50.8%
Total	1,684,848	7.3%	21,516,059	92.7%	23,200,906	100.0%
Seat belt use						
Belted	1,327,539	6.8%	18,210,771	93.2%	19,538,310	91.6%
Unbelted	253,601	14.1%	1,545,673	85.9%	1,799,274	8.4%
Total	1,581,140	7.4%	19,756,444	92.6%	21,337,584	100.0%
Ejection						
Ejected	88,119	67.5%	42,521	32.5%	130,640	0.6%
Not Ejected	1,591,485	6.9%	21,448,651	93.1%	23,040,136	99.4%
Total	1,679,604	7.2%	21,491,172	92.8%	23,170,776	100.0%
Fatality						
Fatal	31,687	29.6%	75,258	70.4%	106,945	0.5%
Not Fatal	1,653,172	7.2%	21,444,711	92.8%	23,097,883	99.5%
Total	1,684,859	7.3%	21,519,969	92.7%	23,204,828	100.0%
b) Injured Occupants Only						
	Rollover		Non-Rollover		Total	
Group	R	R/N	NR	NR/N	N	N/Nall
Sex						
Female	12,727	24.2%	39,893	75.8%	52,620	47.4%
Male	18,509	31.7%	39,911	68.3%	58,420	52.6%
Total	31,236	28.1%	79,804	71.9%	111,040	100.0%
Seat belt use						
Belted	12,619	21.8%	45,243	78.2%	57,863	65.2%
Unbelted	13,499	43.7%	17,421	56.3%	30,920	34.8%
Total	26,118	29.4%	62,664	70.6%	88,783	100.0%
Ejection						
Ejected	9,963	57.6%	7,338	42.4%	17,300	15.7%
Not Ejected	21,078	22.6%	72,139	77.4%	93,218	84.3%
Total	31,041	28.1%	79,477	71.9%	110,518	100.0%
Fatality						
Fatal	5,724	22.6%	19,597	77.4%	25,321	22.8%
Not Fatal	25,512	29.8%	60,207	70.2%	85,719	77.2%
Total	31,236	28.1%	79,804	71.9%	111,040	100.0%

**Table 4:** (a) Proportions and relative risks for all exposed occupants, and (b) raw counts, weighted counts, proportions and relative risks for occupants whose injuries were likely sustained inside the vehicle. The top table summarizes the proportion (95th percentile confidence interval) of each injury type experienced in rollover crashes (top row) and the relative risk (95th percentile confidence interval) of experiencing each injury type in a rollover crash compared to a non-rollover crash for all occupants (first row of relative risks) and for subgroups of female/male, belted/unbelted, and ejected/not ejected occupants (bottom pairs of rows of relative risks). The bottom table also includes the raw and weighted counts for the sub-analysis of occupants whose injuries were likely sustained inside the vehicle

a) All Exposed Occupants												
Injury Group	VI		CI		VCI		All-VI		All-CI		All-Injuries	
	Vertebral Injuries only		Cord injuries		Separate VI and CI		(VI + VCI)		(CI + VCI)		(VI + CI + VCI)	
Proportion in rollover (%)	28.8%	(20.0–37.6)	21.0%	(14.4–27.6)	27.5%	(5.8–58.3)	28.8%	(20.1–37.4)	22.7%	(10.2–35.2)	28.1%	(20.4–35.9)
Relative Risks												
All Occupants	5.17	(3.43–7.81)	3.40	(2.28–5.09)	4.85	(0.86–27.35)	5.16	(3.44–7.74)	3.75	(1.82–7.74)	5.00	(3.46–7.23)
Females	5.52	(2.38–12.80)	3.69	(1.57–8.64)	2.24	(0.32–15.44)	5.48	(2.37–12.65)	3.47	(1.48–8.13)	5.36	(2.38–12.07)
Males	5.03	(3.71–6.83)	3.03	(1.75–5.25)	4.58	(0.68–30.87)	5.01	(3.63–6.91)	3.47	(1.44–8.35)	4.78	(3.51–6.49)
Belted	3.75	(2.02–6.96)	3.88	(2.33–6.46)	8.56	(1.54–47.73)	3.82	(2.05–7.11)	4.61	(2.16–9.85)	3.83	(2.12–6.91)
Unbelted	4.74	(2.15–10.49)	3.60	(0.99–13.11)	8.15	(0.93–71.38)	4.81	(2.20–10.50)	4.49	(1.43–14.09)	4.72	(2.13–10.48)
Ejected	0.89	(0.53–1.51)*	0.28	(0.09–0.89)*	0.06	(0.01–0.58)*	0.70	(0.40–1.22)*	0.14	(0.03–0.64)*	0.66	(0.38–1.13)*
Not Ejected	3.93	(2.34–6.59)	3.07	(1.88–5.01)	11.31	(2.13–59.90)	4.02	(2.41–6.71)	4.01	(2.02–7.95)	3.94	(2.45–6.33)
Fatal	0.85	(0.66–1.10)*	0.24	(0.12–0.48)*	0.15	(0.02–1.09)*	0.83	(0.64–1.09)*	0.23	(0.12–0.47)*	0.69	(0.54–0.90)*
Not Fatal	5.41	(3.38–8.68)	7.06	(3.71–13.44)	5.77	(0.84–39.47)	5.42	(3.43–8.57)	6.53	(2.29–18.58)	5.50	(3.55–8.52)
b) Occupants Whose Injuries Likely Occurred Inside the Vehicle												
Injury Group	VI		CI		VCI		All-VI		All-CI		All-Injuries	
	Vertebral Injuries only		Cord injuries		Separate VI and CI		(VI + VCI)		(CI + VCI)		(VI + CI + VCI)	
Raw counts	147		15		7		154		22		169	
Weighted data	16,417	(7,230 − 25,603)	747	(58 − 1,435)	549	(0–1,384)	16,965	(7,509 − 26,422)	1,296	(0–2,620)	17,712	(8,044 − 27,380)
Proportion in rollover (%)	16.7%	(10.3–23.1)	8.0%	(0.1–15.8)	17.1%	(0.0–45.9)	16.7%	(10.3–23.1)	10.3%	(0.0–21.3)	16.0%	(10.6–21.3)
Relative Risks												
All Occupants	4.58	(2.87–7.31)	1.98	(0.67–5.82)	4.71	(0.65–34.34)	4.58	(2.86–7.36)	2.62	(0.75–9.12)	4.34	(2.85–6.62)

* Significant difference (*p* < 0.05) between the relative risks of the two subgroups (e.g., fatal vs. not fatal)

**Fig. 1:**
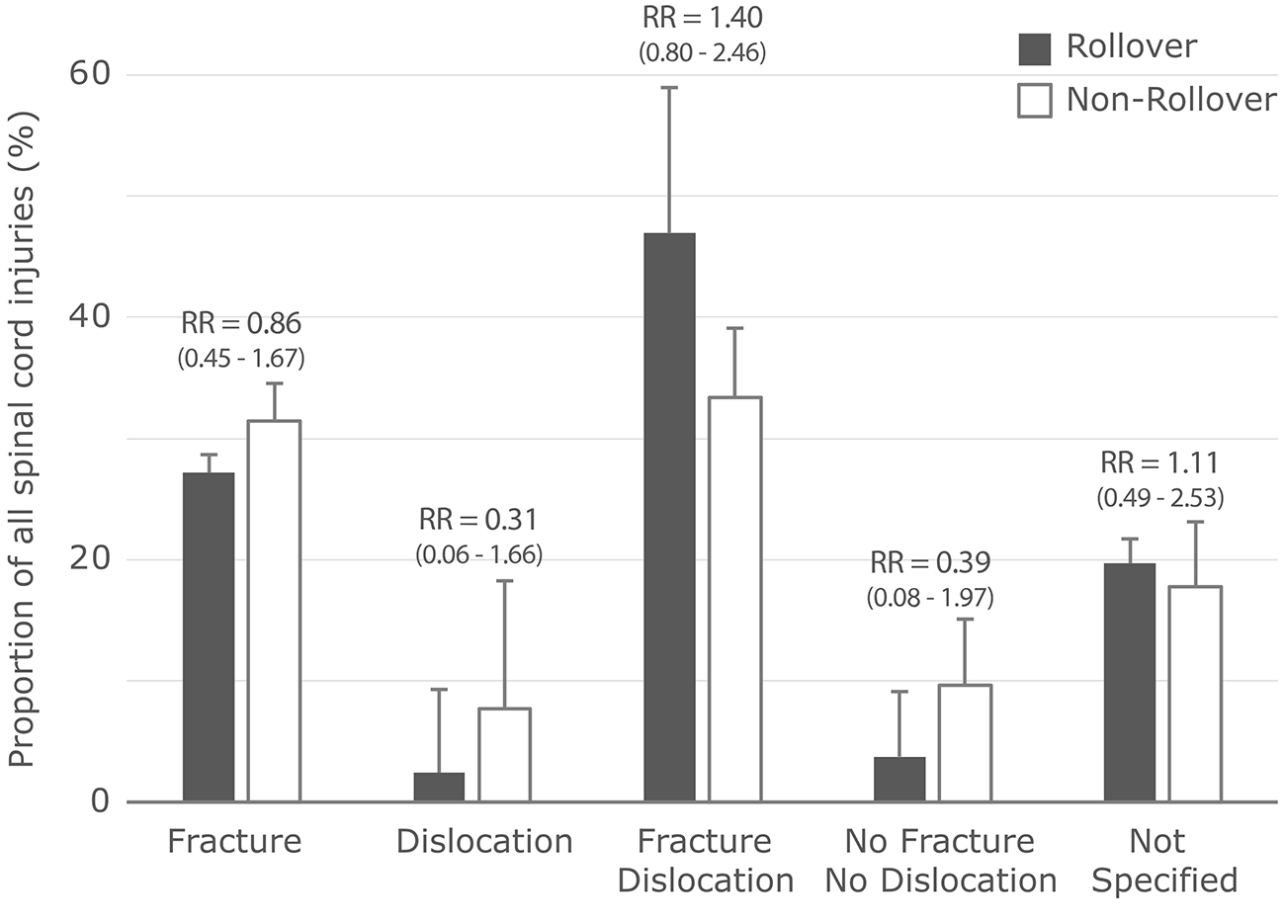
NASS-CDS proportions, standard errors, relative risks (RR), and 95th percentile confidence intervals for RR for all cervical spinal cord injuries (All-CI group) in rollovers (dark bars) and non-rollovers (white bars) stratified by the associated vertebral injury mechanisms (x-axis). All relative risks were not significantly different from one

Of the 2859 cervical spinal cord injuries in rollover crashes, 2188 (77%) had an associated vertebral fracture, dislocation, or fracture/dislocation. This proportion was not significantly different from the rate of cervical fractures and/or dislocations associated with spinal cord injuries in non-rollover crashes (7072/9742 = 73%). Within each type of associated vertebral injury, fractures and fracture/dislocations were most common; however, there were no differences in the relative risks for these associated injuries between rollover and non-rollover crashes (Fig. 1).

### CISS analysis

Of all occupants in our CISS population, 5.5% (0.386 M/7.068 M) or 64,319/year were involved in a rollover crash and 0.6% (0.042 M/7.068 M) or 7001/year experienced a cervical spine injury (Table 5). Like with the NASS-CDS population, about 92.3% (38.8k/42.0k) of the CISS occupants with cervical spine injuries experienced at least one vertebral injury, whereas only 10.8% (4.5k/42.0k) experienced a spinal cord injury (Table 5). Both proportions were similar for rollover occupants (94.3% and 6.0%, respectively) and non-rollover occupants (91.5 and 12.6%, respectively).

**Table 5:** CISS (2017–2022) summary of the (a) raw counts and (b) weighted data for the three combined injury categories, and all exposed occupants. Also shown are the 95th percentile confidence intervals (CI) for the weighted data

a) Raw counts						
Injury Category	Rollover		Non-Rollover		Total	
All-VI (VI + VCI)	50		178		228	
All-CI (CI + VCI)	8		19		27	
All-Injuries (VI + CI + VCI)	57		193		250	
All Exposed Occupants	1,099		11,097		12,196	
b) Weighted data						
Injury Category	Rollover	(95th CI)	Non-Rollover	(95th CI)	Total	(95th CI)
All-VI (VI + VCI)	10,640	(1,533 − 19,747)	28,118	(17,268 − 38,969)	38,758	(28,416 − 49,101)
All-CI (CI + VCI)	679	(0–1,643)	3,867	(1,567–6,167)	4,546	(2,075 − 7,018)
All-Injuries (VI + CI + VCI)	11,286	(1,869 − 20,704)	30,717	(19,042–42,393)	42,003	(30,540 − 53,468)
All Exposed Occupants	385,915	(309,940 − 461,891)	6,682,172	(5,358,153–8,006,190)	7,068,087	(5,702,665–8,433,509)

For the remaining analyses, the smaller sample of injured occupants from the CISS dataset (12,196 raw and 7.1 M weighted cases, Table 5) compared to the NASS-CDS dataset (51,660 raw and 23.2 M weighted cases, Table 2) generated wider confidence intervals and rendered fewer comparisons statistically significant. This deficiency in the CISS dataset was most apparent in the cord injury group (All-CI, containing only 27 raw cases, Table 5a), which meant that the All-VI and All-Injuries groups yielded similar results. Nevertheless, like with the NASS-CDS population, the proportion of occupants exposed to rollover crashes (5.3–5.5% for CISS, Table 6a) was lower than the proportion of all cervical spine injuries that occurred in rollover crashes (26.9%, CI: 7.5–46.3% for CISS, Table 7a). Also similar to the NASS-CDS data, the same general pattern was present in all of the subpopulations except for the ejected and fatal subpopulations (compare the rollover percentages in Table 6a and 6b).

**Table 6:** CISS (2017–2022) weighted counts and proportions (%) for (a) all exposed occupants (injured and uninjured) and (b) all injured occupants (occupants with cervical spine injuries) separated by their exposure to a rollover or non-rollover crash. Separate analyses are tabulated for cases where sex, seatbelt use, ejection status and fatality information was known. Abbreviations: R = rollover, NR = non-rollover, N = weighted counts, Nall = total weighted counts for each condition. The percentages for the rollover (R/N) and non-rollover (NR/N) conditions for each row sum to 100%, whereas the total column percentages (N/Nall) for each group sum to 100%

a) All Exposed Occupants												
	Rollover				Non-Rollover			Total				
Group	R		R/N		NR	NR/N		N		N/Nall		
Sex												
Female	178,674		4.7%		3,613,069	95.3%		3,791,743		54.2%		
Male	203,241		6.3%		2,998,352	93.7%		3,201,593		45.8%		
Total	381,915		5.5%		6,611,421	94.5%		6,993,336		100.0%		
Seat belt use												
Belted	300,689		5.0%		5,687,375	95.0%		5,988,064		90.4%		
Unbelted	56,343		8.9%		577,188	91.1%		633,531		9.6%		
Total	357,032		5.4%		6,264,563	94.6%		6,621,595		100.0%		
Ejection												
Ejected	4,727		66.7%		2,361	33.3%		7,088		0.1%		
Not Ejected	366,229		5.2%		6,667,833	94.8%		7,034,062		99.9%		
Total	370,956		5.3%		6,670,194	94.7%		7,041,150		100.0%		
Fatality												
Fatal	4,868		17.7%		22,666	82.3%		27,534		0.4%		
Not Fatal	381,048		5.4%		6,659,505	94.6%		7,040,553		99.6%		
Total	385,916		5.5%		6,682,171	94.5%		7,068,087		100.0%		
b) Injured Occupants Only												
	Rollover				Non-Rollover			Total				
Group	R		R/N		NR	NR/N		N		N/Nall		
Sex												
Female		8,022*	34.4%	15,280			65.6%		23,302		55.5%	
Male		3,265	17.5%	15,437			82.5%		18,702		44.5%	
Total		11,287	26.9%	30,717			73.1%		42,004		100.0%	
Seat belt use												
Belted		8,049	31.2%	17,746			68.8%		25,795		66.2%	
Unbelted		2,533	19.2%	10,663			80.8%		13,196		33.8%	
Total		10,582	27.1%	28,409			72.9%		38,991		100.0%	
Ejection												
Ejected		650	68.3%	301			31.7%		951		2.3%	
Not Ejected		10,494	25.7%	30,374			74.3%		40,868		97.7%	
Total		11,144	26.6%	30,675			73.4%		41,819		100.0%	
Fatality												
Fatal		660	10.9%	5,398			89.1%		6,058		14.4%	
Not Fatal		10,627	29.6%	25,319			70.4%		35,946		85.6%	
Total		11,287	26.9%	30,717			73.1%		42,004		100.0%	

* One of the 22 raw female cases had a weight of 3270

Compared to occupants involved in non-rollover crashes, the relative risks from the CISS dataset for cervical spine injuries in rollovers were 6.36 (2.11–19.21) for the All-VI group, 3.04 (0.64–14.41) for the All-CI group, and 6.36 (2.21–18.33) for the All-Injuries group (top row of relative risks in Table 7). This pattern of relative risks was also present in the subpopulations (Table 7), albeit fewer of these relative risks were significantly different from one in the CISS data than in the NASS-CDS data. Like the NASS-CDS analysis, female and male occupants, belted and unbelted occupants, non-ejected occupants, and non-fatally injured occupants in the CISS data were more likely to sustain vertebral injuries (All-VI group) in rollovers than in non-rollovers, and ejected occupants and fatally injured occupants were neither more nor less likely to sustain vertebral injuries in rollovers than in non-rollovers (Table 7). With respect to cord injuries (All-CI group) in the CISS data, the average relative risk resembled the pattern in the NASS-CDS data, but none of the CISS-based relative risks were significantly different from unity (Table 7). The relative risks calculated from the NASS-CDS and CISS data were not significantly different.

Our analysis of how different types of vertebral injuries combine with spinal cord injuries revealed that around 90% (615/697 occupants) of cord injuries in rollover occupants had associated fracture-dislocations (60.9%, 60.3–61.4%) and fractures (29.7%, 29.3–30.2%) (Table S1 in the Supplemental Materials). Due to the paucity of CISS spinal cord injury data (only 27 raw All-CI cases), we could not resolve within- and between-group differences in this sub-analysis. Similar to NASS-CDS, our sub-analysis that filtered for non-ejected occupants whose injuries likely occurred inside the rolling vehicle saw proportions (2.1%, 860/42,004 occupants) and relative risks (1.66, 0.55–4.99) diminish but had large confidence limits (Table S2 in the Supplemental Materials).

**Table 7:** CISS (2017–2022) proportions and relative risks for all exposed occupants. Table summarizes the proportion (95th percentile confidence interval) of each injury type experienced in rollover crashes (top row) and the relative risk (95th percentile confidence interval) of experiencing each injury type in a rollover crash compared to a non-rollover crash for all occupants (first row of relative risks) and for subgroups of female/male, belted/unbelted, and ejected/not ejected occupants (bottom pairs of rows of relative risks)

All Exposed Occupants						
Injury Group	All-VI		All-CI		All-Injuries	
	(VI + VCI)		(CI + VCI)		(VI + CI + VCI)	
Proportion in rollover (%)	27.45%	(7.07–47.84)	14.94%	(0.00–31.71)	26.87%	(7.48–46.26)
Relative Risks						
All Occupants	6.55	(2.18–19.71)	3.04	(0.64–14.41)	6.36	(2.21–18.33)
Females	11.52	(2.64–50.31)	-	-	10.62	(2.49–45.27)
Males	2.75	(1.38–5.48)	4.56	(0.92–22.63)	3.12	(1.68–5.80)
Belted	9.12	(2.32–35.82)	2.40	(0.44–13.12)	8.58	(2.22–33.13)
Unbelted	2.23	(1.13–4.41)	2.94	(0.43–20.06)	2.43	(1.25–4.74)
Ejected	1.17	(0.29–4.73)*	0.71	(0.03–15.22)	1.08	(0.26–4.40)*
Not Ejected	6.45	(2.05–20.26)	3.06	(0.63–14.83)	6.29	(2.11–18.79)
Fatal	0.59	(0.17–2.01)*	0.27	(0.01–4.86)	0.57	(0.16–1.99)*
Not Fatal	7.49	(2.41–23.23)	4.01	(0.82–19.59)	7.34	(2.47–21.81)

* Significant difference (*p* < 0.05) between the relative risks of the two subgroups (e.g., fatal vs. not fatal)

- There were no instances of females in rollovers with spinal cord injury

## Discussion

The overall goal of this study was to examine how different types of cervical spine injuries vary with different occupant-related factors in rollover and, for comparison purposes, non-rollover crashes. To achieve this goal, we relied on weighted crashes from the NASS-CDS database for the years 2005–2015 and the CISS database for the years 2017–2022, and we focused our attention on occupants with vertebral or spinal cord injuries in the cervical spine. Across both databases, we found that most occupants with cervical spine injuries in rollover crashes involved only vertebral injuries (91 to 94%), and that spinal cord injuries occurred in only 6.0 to 9.1% of all occupants with cervical vertebral column injuries associated with rollover crashes. We also found that cervical spine injuries involving vertebral fractures were 5.2 times (NASS-CDS) to 6.4 times (CISS) more likely to occur in rollover crashes than in non-rollover crashes. Even for the subpopulations related to sex, seatbelt use, ejection and fatality, the relative risks in our CISS analysis remained similar to or greater than the relative risks in our NASS-CDS analysis despite a presumably safer fleet of vehicles in the CISS database. Given the high societal costs associated with cervical spine injuries—especially spinal cord injuries—these findings highlight the importance of developing countermeasures aimed at either preventing rollover crashes from happening or preventing cervical spine injuries in rollover crashes that do happen.

Our CISS sample was smaller than our NASS-CDS sample and thus yielded wider confidence intervals, especially for the spinal cord injury group. Interestingly, while relative risks in the CISS and NASS-CDS analyses were similar, annual injury rates dropped disproportionally in our CISS data (from 10,095 cervical spine injuries per year in NASS-CDS to 7,001 cervical spine injuries per year in CISS). To determine if this drop was due to vehicle improvements or solely due to the narrower model and crash year filter in the CISS analysis, we reran the NASS-CDS analyses using 2010–2015 data and MODELYR ≥ 2002 to match the ranges used in the CISS analysis. Annual NASS-CDS case numbers decreased to 6,229 injuries per year, and relative risks were lower than in the original NASS-CDS analysis (Table S3 in the Supplemental Materials). One possible explanation for these findings is that safety improvements in the newer fleet may be offset by an increase in the number of vehicles more prone to rollovers (e.g., SUVs and trucks) (Statista Market and Insights 2023). In partial support of this explanation, a post hoc analysis revealed that 21.7% of occupants with cervical spine injury in the NASS-CDS dataset were in utility vehicles, whereas 50.6% of occupants with cervical spine injuries in the CISS dataset were in utility vehicles. Further work exploring the interaction of safety improvements and fleet composition is warranted.

Although rollovers have a higher risk than non-rollovers of causing cervical vertebral injuries (NASS-CDS and CISS data) and spinal cord injuries (NASS-CDS data only), these increased risks were not significantly different between females and males (*p* > 0.60 in both NASS-CDS and CISS). This finding is consistent with previous rollover-specific research (Funk et al. 2012; Ivarsson et al. 2015). Sex, however, is a complex variable that combines many intrinsic factors (e.g., height, weight, vertebral size, vertebral tolerance to load, neck length, neck strength, etc.) (Ezra et al. 2017; Pan et al. 2018; Vasavada et al. 2008) that could interact with various extrinsic factors (e.g., seat geometry, seat belt fit, occupant compartment geometry, etc.) to wash out specific sex differences that potentially could be leveraged to improve occupant protection. Further work is needed to explore the potential interaction of these factors and whether they influence cervical injury outcomes differently for females and males.

Similar findings were observed in relation to seatbelts, where the relative risks of sustaining different cervical spine injuries in rollover and non-rollover crashes were not significantly different for belted and unbelted occupants (NASS-CDS: *p* > 0.64, CISS: *p* > 0.44). Many prior studies have shown that unbelted occupants are at higher risk for injury than belted occupants (Bedewi et al. 2004; Funk et al. 2012; Moore, 2005; Parenteau and Viano 2014), and our data aligns with prior findings that proportionally more cervical spine injuries occur in rollovers than in non-rollovers (Parenteau and Viano 2014; Yadollahi et al. 2016; Yoganandan et al. 1989a); however, our data also shows that there is no specific category of cervical spine injury that is disproportionately more or less likely in rollovers than in non-rollovers between belted and unbelted occupants. This finding is perhaps surprising given that unbelted occupants include virtually all ejected occupants, who have much higher risks of sustaining a cervical spine injury than those who are not ejected. For instance, in a post hoc analysis of rollovers in the NASS-CDS data, ejected occupants were 8.8 (6.2–12.3) times more likely to have a vertebral injury (All-VI group) than non-ejected occupants, and in non-rollovers, ejected occupants were 50 (23–110) times more likely to have a vertebral injury (All-VI group) than non-ejected occupants. In absolute terms, however, the number of ejected occupants is small (< 1% of all exposed occupants, with about two-thirds occurring in rollover crashes; Tables 3 and 7), and therefore their influence on the relative risk values may be too small to affect the overall relative risks.

Within ejected occupants in our NASS-CDS analysis, the relative risks for cord-related injuries were significantly less than one (RR = 0.14, Table 4), indicating that ejected occupants in rollovers were less likely to have a spinal cord injury than ejected occupants in non-rollover crashes. The corresponding relative risk for the CISS analysis was also less than one, but the low sample size rendered it not significant. A post hoc analysis of the NASS-CDS data revealed that in rollover crashes, ejected occupants were 5.4 (1.7–17.1) times more likely to have a spinal cord injury (All-CI group) than non-ejected occupants, and in non-rollover crashes, ejected occupants were 157 (41–604) times more likely to have a spinal cord injury than non-ejected occupants. From a spinal cord injury perspective, this finding may imply that it is much worse to be ejected from a non-rollover crash than a rollover crash. One possible explanation for this phenomenon is that ejected occupants in non-rollover crashes experience more severe collisions than non-ejected occupants. Indeed, a follow-up calculation showed that the speed change (DVTOTAL) in non-rollover crashes was 36.6 ± 2.5 km/h (mean ± SE) for ejected occupants compared to 19.8 ± 0.1 km/h for non-ejected occupants. A similar comparison for rollover crashes is less meaningful because ejection is likely related more to angular roll speed and vehicle configuration, window damage and deformation that allows for ejection than to the linear speed change of any single impact during a rollover event, and roll speed is not recorded in the NASS-CDS database. Nevertheless, these findings highlight that ejection is a serious risk for cervical spinal cord injury in both rollover and non-rollover crashes.

We found that 18% (5,724/31,236 in Table 3b) of NASS-CDS rollover occupants with cervical spine injuries were fatally injured, a rate that is almost 10 times higher than the general fatality rate in rollovers (1.9%, 31,687/1,684,859 in Table 3a). In non-rollovers, however, 24% (19,597/79,804 in Table 3b) of occupants with cervical spine injuries were fatally injured, a rate that is 70 times higher than the general fatality rate in non-rollovers (0.35%, 75,258/21,519,969 in Table 3a). CISS data followed a similar trend but with lower overall fatality rates. In rollovers, 5.9% (31,687/1,684,859 in Table 6b) of occupants with cervical spine injuries were killed, about 5 times the general fatality rate of 1.3% (4,868/385,916 in Table 6a), whereas in non-rollovers, 17.6% (5,398 /30,717 in Table 6b) of occupants with cervical spine injuries were killed, about 53 times the general fatality rate of 0.3% (22,666 /6,682,171 in Table 6a). An explanation for why the fatality rate of cervical-spine-injured occupants is higher in non-rollovers than rollovers cannot be reached here due to missing injury information for many fatal cases in both NASS-CDS and CISS.

Even though we restricted our CISS database analyses to vehicles manufactured in and after 2010, the relative risks and rollover proportions in the CISS analysis were similar or greater than those in the NASS-CDS analysis (compare Table 4a and Table 7). Since 2010, the vehicle fleet underwent major advances in safety systems, e.g., the introduction of roof strength (FMVSS No. 216a, 2009) and ejection mitigation (FMVSS No. 226, 2011) legislation. As might be expected, there was a 5.6- to 6.9-fold reduction in ejections for both the exposed and injured populations (Tables 3 and 6) in rollovers and non-rollovers. However, the relative risks for vertebral injuries remained high in the later model vehicles included in our CISS analysis. Advances in passive safety may have been successful in reducing the number of injured occupants per year, however, rollover-specific technology, like roof airbag systems and roof designs (Halldin et al. 2000; Heudorfer et al. 2005; Lee 2021) may be needed to target rollover-specific occupant impact mechanisms that have not been addressed by existing technology.

Many cadaveric experiments (Alem et al. 1984; McElhaney et al. 1983; Nightingale et al. 1991; Pintar et al. 1989; Yoganandan et al. 1989b) have sought to quantify injury thresholds and responses of the cervical spine to axial compression but have failed to consistently reproduce real-world injury patterns that occur in automotive rollovers (Foster et al. 2012). Nevertheless, clinically relevant cervical spine fractures are consistently caused by headfirst impacts that compress the cervical spine in cadaver experiments (Nightingale et al. 1996a, b, 1997; Saari et al. 2011). Moreover, these fractures occur early (2 to 19 ms after head contact), well before any significant head motion develops and well before reflex muscle activation could develop in living humans. The early occurrence of these fractures indicates that they are likely caused by local flexion and extension buckling of the cervical spine due to compression rather than excessive flexion, rotation or extension of the head and neck (Nightingale et al. 2019). Posterior injuries and other injuries associated with spinal loads absent axial compression like the “Clay-shoveler’s” fracture were found to be rare in our dataset (~ 8% of injuries in CISS) and in previous studies examining both rollovers and non-rollovers (Foster et al. 2012).

Here, we sought to find injury patterns unique to rollovers, as the kind of cervical spine injury an occupant sustains may reveal the underlying initial conditions of the headfirst impact leading to axial loading of the neck (Winkelstein and Myers 1997). Despite the rare occurrence of cervical spinal cord injuries, we found that 73 and 77% of cervical spinal cord injuries in the NASS-CDS dataset (50 and 90% in CISS) had associated fractures or fracture-dislocations in non-rollover and rollover occupants, respectively. These results suggest that there is a strong relationship between spinal cord injuries and the presence of fractures and/or fracture-dislocations, a finding that is consistent with clinical evidence of the prevalence of these injuries in both motor-vehicle collisions and other contexts (Sekhon and Fehlings 2001). Future efforts aimed at preventing vertebral and spinal cord injuries that prioritize reducing cervical spine fractures will simultaneously reduce cervical spinal cord injuries as well.

The generalizability of our analysis is limited by some of our methods. Our analysis included injuries that may have occurred before or after the rollover itself. Previous researchers (Bose et al. 2011; Funk et al. 2012; McMurry et al. 2016) saw a large decrease in cases when they tried to restrict their data to pure rollovers without planar impacts. Bose et al. (2011) proposed that ignoring the contribution of associated planar impacts might alter vehicle intrusion and the occupant’s initial position, two factors that could affect injury risk. When we attempted to isolate only those injuries that occurred during the rollover and also excluded planar impacts (Table 4b), we found similar (*p* > 0.36), albeit reduced, relative risks. One limitation of using the NASS-CDS and CISS databases is a lack of in-depth injury documentation that could help further ascertain injury patterns unique to rollovers. While data from the CIREN includes imaging and autopsy data, its small sample size is insufficient to perform the analyses described in this study. Also, we chose to differentiate cervical spine injuries based on whether the cord was involved or not. An alternative differentiation could be based on benign and clinically relevant injuries, however both NASS-CDS and CISS rely on AIS codes that are not well suited for such differentiation.

Our study was aimed at describing the occupant cohort who sustained cervical spine injuries and to contrast them between rollovers and non-rollovers. We used occupant variables, like sex, belt status, ejection, and fatality to describe how these sub-groups were correlated with injury outcome. We did not repeat prior analyses on crash-related factors known to influence rollover injury severity, like the number of rolls, the extent of roof crush, roll direction, and the occupant’s seat position (Hu 2007; Hu et al. 2007; McMurry et al. 2016; Padmanaban 2005; Parenteau 2001; Viano and Parenteau 2018). While we acknowledge the importance of these other crash-related factors, combining these other factors with our variables of interest led to sample sizes that were too small to yield meaningful insights.

**Fig. 2:**
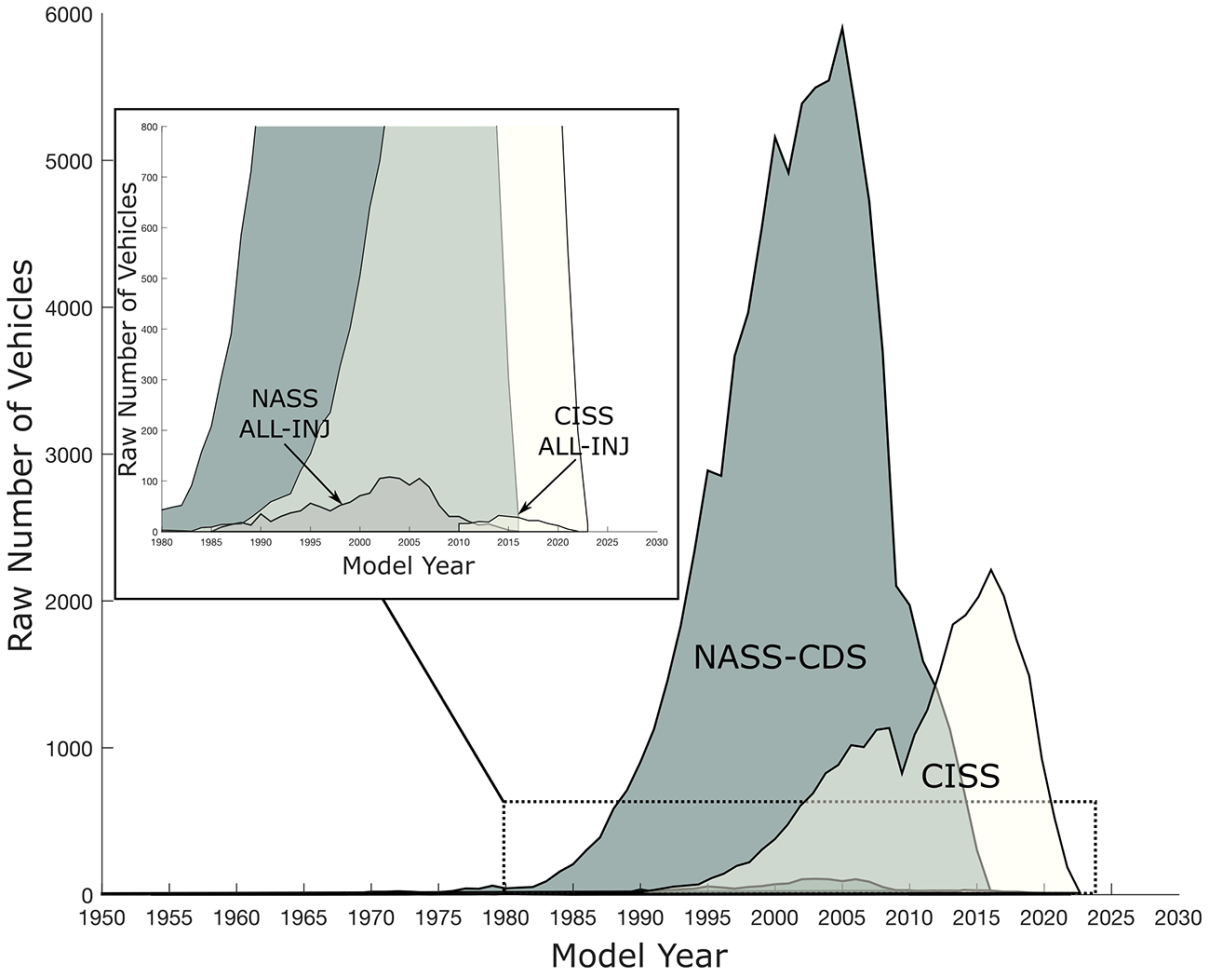
Model years of crash-involved vehicles for NASS-CDS and CISS. The raw numbers of vehicles per model year are shown for NASS-CDS (2005–2015) and CISS (2017–2022) data. The inset graph shows a zoomed in view of the All-Injuries groups in both analyses

Our NASS-CDS analysis relied on data from the last 11 years of this database and included vehicle model years 1985 to 2016. Safety improvements to vehicles since 2015 were not captured in our NASS-CDS analyses. In particular, the increased use of side curtain airbags in the vehicle fleet (which is not recorded in NASS) could affect the relative risks we report here (Li et al. 2018). Despite a recent trend of using combined CISS and NASS-CDS data to gain statistical power (Craig et al. 2024; Viano and Parenteau 2023), our approach enabled us to see the persistent high rates of cervical spine injuries in rollovers. Moreover, a comparison of vehicle model years between the NASS-CDS and CISS datasets (Fig. 2) revealed substantial overlap between the entire fleets in the two databases, but relatively little overlap in our injured population due to our model-year filters.

## Conclusions

In summary, we sought to characterize the relative frequencies and relative risks of different types of cervical spine injuries in rollover and non-rollover crashes. Despite accounting for only 7.3% of collisions in NASS-CDS (5.5% of collisions in CISS), rollovers generated 23% (15%) of cervical spinal cord injuries and 28% (28%) of cervical vertebral injuries. The relative risks for cervical vertebral and spinal cord injuries were 3.4 to 5.2 times (CISS: 3.0 to 6.6 times) higher in rollover than in non-rollover crashes. These relative risks were similar for male and female occupants, belted and unbelted occupants, non-ejected occupants, and non-fatal occupants. Despite advancements in vehicle safety technology in the CISS crashes, these proportions and risks remained disproportionally high for rollover occupants across both databases. There was no specific category of cervical spine injury that was more or less likely in rollovers, although vertebral injuries were more common (NASS-CDS: 91.5%, CISS: 94.3%) than spinal cord injuries (11.5%, 6.0%). Lastly, these findings suggest that research focused on preventing cervical vertebral fractures will prevent serious and debilitating injuries to the spinal column while also effectively preventing most cervical spinal cord injuries.

### Electronic supplementary material

Below is the link to the electronic supplementary material.

Supplementary Material 1

## Data Availability

The NASS-CDS and CISS datasets generated and/or analysed during the current study are available in the National Highway Traffic Safety Administration’s repository, under https://www.nhtsa.gov/file-downloads?p=nhtsa/downloads/.

## Footnotes

1 CIREN (Crash Injury Research Engineering Network) is a database by the National Highway Traffic Safety Administration that includes crash investigation data, while providing multidisciplinary analyses of medical and engineering evidence to determine injury causation (“Crash Injury Research | NHTSA,” n.d.)

## Abbreviations

AIS: Abbreviated injury scale
ATD: Anthropometric test device
CIREN: Crash injury research engineering network
CISS: Crash investigation sampling system
FMVSS: Federal motor vehicle safety standard
NASS-CDS: National automotive sampling system – crashworthiness data system
NHTSA: National highway traffic safety administration
PSU: Primary sampling unit
RR: Relative risk
SCI: Spinal cord injury

## References

[CR1] Alem N;, Nusholtz G, Melvin J. 1984. Head and neck response to axial impacts, in: Proceedings of the 28th Stapp Car Crash Conference. Presented at the Proceedings of the 28th Stapp Car Crash Conference, Chicago, Illinois, pp. 275–288.

[CR2] Altman DG, Bland JM (2003). Interaction revisited: the difference between two estimates. BMJ.

[CR3] Association for the Advancement of Automotive Medicine. 2008. Abbreviated Injury Scale 2005 - Update 2008.

[CR4] Bahling GS, Bundorf RT, Kaspzyk GS, Moffatt EA, Orlowski KF, Stocke JE. 1990. Rollover and drop tests - the influence of roof strength on injury mechanics using belted dummies. Soc Automot Eng 902314.

[CR5] Bedewi PG, Godrick DA, Digges KH, Bahouth GT (2004). An investigation of occupant injury in rollover: NASS-CDS analysis of injury severity and source by rollover attributes. Prog Technol.

[CR6] Berkowitz M. Spinal cord Injury: an analysis of medical and social costs. Demos Medical Publishing; 1998.

[CR7] Bilston LE, Clarke EC, Brown J (2011). Spinal injury in car crashes: crash factors and the effects of occupant age. Inj Prev.

[CR8] Bose D, Kerrigan JR, Foster JB, Crandall JR, Tobaru S (2011). Planar impacts in rollover crashes: significance, distribution and injury epidemiology. Ann Adv Automot Med Annu Sci Conf Assoc Adv Automot Med Assoc Adv Automot Med Sci Conf.

[CR9] Burns SP, Kaufman RP, Mack CD, Bulger E (2010). Cost of spinal cord injuries caused by rollover automobile crashes. Inj Prev.

[CR10] Cooper ER, Croteau JJ, Parenteau C;, Toglia A. Head excursion of seat belted cadaver, volunteers and hybrid III ATD in a dynamic/static rollover fixture. Volume 973347. SAE Tech. Pap; 1997.

[CR11] Craig MJ, Liu C, Zhang F, Enriquez J (2024). Sex-based differences in odds of motor vehicle crash injury outcomes. Accid Anal Prev.

[CR12] Crash Injury Research | NHTSA [WWW Document]. n.d. URL https://www.nhtsa.gov/research-data/crash-injury-research (accessed 4.19.24).

[CR13] Digges KH. 2002. Summary Report of Rollover Crashes. Tech. Reporrt FHWANHTSA Natl. Anal. Cent. Wash. DC 32.

[CR14] Ezra D, Masharawi Y, Salame K, Slon V, Alperovitch-Najenson D, Hershkovitz I (2017). Demographic aspects in cervical vertebral bodies’ size and shape (C3–C7): a skeletal study. Spine J.

[CR15] Fakharian E, Mohammadzadeh M, Saberi HR, Fazel MR, Rejali M, Akbari H, Mirzadeh AS, Mohammadzadeh J (2017). Spinal injury resulting from car accident: focus to prevention. Asian J Neurosurg.

[CR16] Foster JB, Kerrigan JR, Nightingale RW, Funk JR, Cormier JM, Bose D, Sochor MR, Ridella SA, Ash JH, Crandall JR. 2012. Analysis of cervical spine injuries and mechanisms for CIREN rollover crashes, in: Proceedings of the International Research Council on the Biomechanics of Injury Conference. pp. 61–75.

[CR17] Funk JR, Cormier JM, Manoogian SJ (2012). Comparison of risk factors for cervical spine, head, serious, and fatal injury in rollover crashes. Accid Anal Prev.

[CR18] Gloeckner DC, Moore TLA, Steffey D, Bare C, Corrigan CF (2006). Implications of Vehicle Roll Direction on Occupant Ejection and Injury Risk. Annu Proc Assoc Adv Automot Med.

[CR19] Halldin PH, Brolin K, Kleiven S, von Holst H, Jakobsson L, Palmertz C (2000). Investigation of conditions that affect Neck Compression- Flexion injuries using Numerical techniques. Stapp Car Crash J.

[CR20] Heudorfer B, Breuninger M, Karlbauer U, Kraft M;, Maidel J. 2005. A concept study to provide enhanced protection for head and neck in case of rollover. Presented at the International Technical Conference on the Enhanced Safety of Vehicles.

[CR21] Hu J (2007). Neck Injury mechanism in Rollover crashes - a systematic Approach for improving Rollover Neck Protection.

[CR22] Hu J, Chou CC, Yang KH, King AI. 2007. A weighted logistic regression analysis for predicting the odds of head/face and neck injuries during rollover crashes. Annu. Proc. Assoc. Adv. Automot. Med. Assoc. Adv. Automot. Med. 51, 363–379. PMC3217522 18184502

[CR23] Ivarsson J, Poplin G, McMurry T, Crandall J, Kerrigan J (2015). Occupant injury in rollover crashes - contribution of planar impacts with objects and other vehicles. Accid Anal Prev 85 acs.

[CR24] Jehle D, Kuebler J, Auinger P (2007). Risk of injury and fatality in single vehicle rollover crashes: danger for the front seat occupant in the outside arc. Acad Emerg Med off J Soc Acad Emerg Med.

[CR25] Lee DO. Roof airbag for vehicles and control method to deploy same. US20210245692A1; 2021.

[CR26] Li H, Jiang C, Cui D, Lu S. The effects of Curtain Airbag on Occupant Kinematics and Injury Index in Rollover Crash. Appl Bionics Biomech. 2018;2018(4980413). 10.1155/2018/4980413. 10.1155/2018/4980413 PMC5885342 29765463

[CR27] Ma VY, Chan L, Carruthers KJ (2014). Incidence, prevalence, costs, and impact on disability of common conditions requiring rehabilitation in the United States: stroke, spinal cord injury, traumatic brain injury, multiple sclerosis, osteoarthritis, rheumatoid arthritis, limb loss, and back pain. Arch Phys Med Rehabil.

[CR28] Mandell SP, Kaufman R, Mack CD, Bulger EM (2010). Mortality and injury patterns associated with roof crush in rollover crashes. Accid Anal Prev.

[CR29] McDowell MA, Ogden FCD, Flegal CL, K.M. Anthropometric Reference Data for Children and adults: United States, 2003–2006. National Health Statistics Reports; 2008. 25585443

[CR30] McElhaney JH, Paver JG, McCrackin HJ, Maxwell GM. 1983. Cervical spine compression responses, in: Proceedings of the 27th Stapp Car Crash Conference. Presented at the Proceedings of the 27th Stapp Car Crash Conference, San Diego, California, pp. 163–178.

[CR31] McMurry TL, Bose D, Ridella SA, Eigen AM, Crandall JR, Kerrigan JR (2016). Epidemiology of moderate-to-severe injury patterns observed in rollover crashes. Accid Anal Prev.

[CR32] Moffatt E, Hare B, Hughes R, Lewis L, Iiyama H, Curzon A, Cooper E (2003). Head excursion of restrained human volunteers and hybrid III dummies in steady state rollover tests. Annu Proc Assoc Adv Automot Med.

[CR33] Moore T, Ramachandran SD, Corrigan K. C., 2005. Biomechanical Factors and Injury Risk in High Severity Rollovers. 49th Annu. Proc. Assoc. Adv. Automot. Med. 133–150. PMC3217444 16179145

[CR34] Mynatt M, Brophy J. 2017. Improved Field Measurements in NHTSA’s CISS Program. Presented at the 25th International Technical Conference on the Enhanced Safety of Vehicles (ESV)National Highway Traffic Safety Administration.

[CR35] National Center for Statistics and Analysis. 2020. Traffic Safety Facts 2018: A Compilation of Motor Vehicle Crash Data (No. DOT HS 812 981).

[CR36] National Spinal Cord Injury Statistical Center. Spinal cord injury (SCI) facts and figures at a glance. University of Alabama at Birmingham; 2020.

[CR41] Nightingale RW, Myers BS, McElhaney JH, Richardson WJ, Doherty BJ (1991). The influence of end condition on human cervical spine injury mechanisms. Soc Automot Eng Trans Pap.

[CR39] Nightingale RW, McElhaney JH, Richardson WJ, Best TM, Myers BS (1996). Experimental impact injury to the cervical spine: relating motion of the head and the mechanism of injury. J Bone Jt SurgeryAmerican Vol.

[CR40] Nightingale RW, McElhaney JH, Richardson WJ, Myers BS (1996). Dynamic response of the head and cervical spine to axial impact loading. J Biomech.

[CR38] Nightingale RW, McElhaney JH, Camacho DL, Kleinberger M, Winkelstein BA, Myers BS. 1997. The dynamic responses of the cervical spine: buckling, end conditions, and tolerance in compressive impacts, in: Proceedings of the 41st Stapp Car Crash Conference. Presented at the Proceedings of the 41st Stapp Car Crash Conference, Lake Buena Vista, Florida, pp. 451–472.

[CR37] Nightingale RW, Bass CR, Myers BS (2019). On the relative importance of bending and compression in cervical spine bilateral facet dislocation. Clin Biomech.

[CR42] Padmanaban J, H. S. 2005. Occupant Injury Experience in Rollover Crashes: An In-Depth Review of NASS/CDS Data. 49th Annu. Proc. Assoc. Adv. Automot. Med. 103–118. PMC3217447 16179143

[CR43] Pan F, Arshad R, Zander T, Reitmaier S, Schroll A, Schmidt H (2018). The effect of age and sex on the cervical range of motion - A systematic review and meta-analysis. J Biomech.

[CR44] Parenteau C. Near and Far-Side Adult Front passenger kinematics in a vehicle rollover. SAE Tech. Pap. Ser; 2001.

[CR45] Parenteau CS, Viano DC (2014). Spinal fracture-dislocations and spinal cord injuries in Motor Vehicle crashes. Traffic Inj Prev.

[CR46] Parker DD, Moore RRM, Keefer TLA. R.E., 2007. Rollover Severity and Occupant Protection - A Review of NASS/CDS Data. SAE Tech. Pap. Ser. No 2007-01-0676.

[CR47] Pintar F, Yoganadan N, Sances A. Kinematic and anatomical analysis of the human cervical spinal column under axial loading. SAE Tech. Pap; 1989. p. 831616.

[CR48] Raddin J, Cormier J, Smyth B, Croteau J, Cooper E (2009). Compressive Neck Injury and its relationship to Head Contact and Torso Motion during Vehicle rollovers. SAE Int J Passeng Cars - Mech Syst.

[CR49] Radja G. 2016. National Automotive Sampling System, Crashworthiness Data System – 2015 Analytical User’s Manual 152.

[CR50] Radja GA, Noh EY, Zhang F (2023). Crash Investigation Sampling System 2022 analytical user’s manual. (Report No DOT HS.

[CR51] Ridella S, Eigen A. 2008. Biomechanical Investigation of Injury Mechanisms in Rollover Crashes from the CIREN Database. Presented at the IRCOBI Conference, Bern, Switzerland, pp. 33–47.

[CR52] Saari A, Itshayek E, Cripton PA (2011). Cervical spinal cord deformation during simulated head-first impact injuries. J Biomech.

[CR53] Sekhon LH, Fehlings MG (2001). Epidemiology, demographics, and pathophysiology of acute spinal cord injury. Spine.

[CR54] State Laws [WWW Document], n.d. Car Seats Littles. URL https://csftl.org/state-laws/ (accessed 7.19.21).

[CR55] Statista Market, Insights. 2023. SUVs - United States | Statista Market Forecast [WWW Document]. Statista. URL https://www.statista.com/outlook/mmo/passenger-cars/suvs/united-states (accessed 3.31.23).

[CR56] Stein DM, Kufera JA, Ho SM, Ryb GE, Dischinger PC, O’Connor JV, Scalea TM (2011). Occupant and Crash characteristics for case occupants with cervical spine injuries sustained in Motor Vehicle collisions. J Trauma Acute Care Surg.

[CR57] The Association for Advancement of Automotive Medicine. 2016. Abbreviated Injury Scale 2015.

[CR58] United States Government Accountability Office. 2015. Status of NHTSA’s Redesign of Its Crashworthiness Data System [WWW Document]. URL https://www.gao.gov/assets/gao-15-334.pdf (accessed 7.19.21).

[CR59] Vasavada AN, Danaraj J, Siegmund GP (2008). Head and neck anthropometry, vertebral geometry and neck strength in height-matched men and women. J Biomech.

[CR61] Viano DC, Parenteau CS (2018). Rollover injury in vehicles with high-strength-to-weight ratio (SWR) roofs, curtain and side airbags, and other safety improvements. Traffic Inj Prev.

[CR60] Viano DC, Parenteau CS (2023). Fetal deaths and maternal injury in motor-vehicle crashes using NASS-CDS and CISS field data. Traffic Inj Prev.

[CR62] Viano DC, Parenteau CS, Edwards ML (2007). Rollover injury: effects of near-and far-seating position, belt use, and number of quarter rolls. Traffic Inj Prev.

[CR63] Winkelstein BA, Myers BS (1997). The biomechanics of cervical spine injury and implications for injury prevention. Med Sci Sports Exerc.

[CR64] Yadollahi M, Paydar S, Ghaem H, Ghorbani M, Mousavi SM, Taheri Akerdi A, Jalili E, Niakan MH, Khalili HA, Haghnegahdar A, Bolandparvaz S. 2016. Epidemiology of cervical spine fractures. Trauma Mon 21 3, e33608. 10.5812/traumamon.33608 PMC5124335 27921020

[CR65] Yoganandan N, Haffner M, Maiman DJ, Nichols H, Pintar FA, Jentzen J, Weinshel SS, Larson SJ Jr., A. Epidemiology and injury biomechanics of motor vehicle related trauma to the human spine. SAE Tech. Pap; 1989a. p. 892438.

[CR66] Yoganandan N, Sances A, Pintar F (1989). Biomechanical evaluation of the axial compressive responses of the human cadaveric and manikin necks. J Biomech Eng.

[CR67] Zhang F, Subramanian R, Chen C-L, Noh EY. 2019. Crash Investigation Sampling System: Design Overview, Analytic Guidance, and FAQs (Report No. DOT HS 812 801).

