# Supplementary material for: Cervical vertebral and spinal cord injuries in rollover occupants
Source: Inj Epidemiol. 2024 Jul 3;11:30. doi: 10.1186/s40621-024-00506-4 (PMC11223401; doi:10.1186/s40621-024-00506-4)
Supplement: Supplementary file 1 — Supplementary Material 1 [file 40621_2024_506_MOESM1_ESM.docx]

**SUPPLEMENTAL MATERIALS**

Table S1: SAS output for CISS (2017–2022) for all cervical spinal cord injuries (ALL-CI) for rollover (top) and non-rollover (bottom) occupants*.*

| \| **Data Summary** \| \| \| --- \| --- \| \| Number of Strata \| 10 \| \| Number of Clusters \| 18 \| \| Number of Observations \| 27 \| \| Sum of Weights \| 4546.46265 \| |
| --- | --- | --- | --- | --- | --- | --- | --- | --- | --- | --- |
| \| **SCITYPE1** \| **Frequency** \| **Weighted Frequency** \| **Std Err of Wgt Freq** \| **95% Confidence Limits for Wgt Freq** \| \| **Percent** \| **Std Err of Percent** \| \| --- \| --- \| --- \| --- \| --- \| --- \| --- \| --- \| \| Fractures (Fx) \| 3 \| 413.48188 \| 4.86679 \| 351.64342 \| 475.32035 \| 60.8749 \| 0.2803 \| \| Fracture-Dislocations \| 4 \| 201.93721 \| 0 \| 201.93721 \| 201.93721 \| 29.7302 \| 0.2130 \| \| NFS \| 1 \| 63.81316 \| 0 \| 63.81316 \| 63.81316 \| 9.3949 \| 0.0673 \| \| Total \| 8 \| 679.23226 \| 4.86679 \| 617.39379 \| 741.07072 \| 100.0000 \|  \| |
| 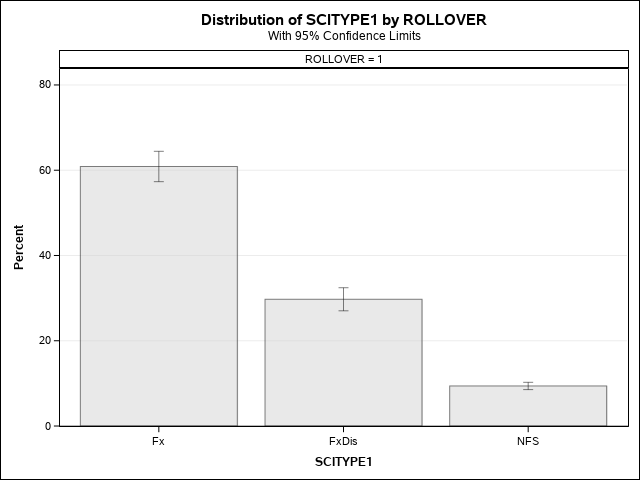 |
| \|  \| **SCITYPE1** \| **Frequency** \| **Weighted Frequency** \| **Std Err of Wgt Freq** \| **95% Confidence Limits for Wgt Freq** \| \| **Percent** \| **Std Err of Percent** \| \| --- \| --- \| --- \| --- \| --- \| --- \| --- \| --- \| --- \| \|  \| Dislocation (Dis) \| 2 \| 214.96027 \| 28.62746 \| 144.91141 \| 285.00913 \| 5.5585 \| 1.6153 \| \|  \| Fractures (Fx) \| 9 \| 1928 \| 788.11651 \| 0 \| 3857 \| 49.8662 \| 14.5608 \| \|  \| NFS \| 6 \| 1581 \| 717.68187 \| 0 \| 3337 \| 40.8791 \| 14.5404 \| \|  \| NoFx/NoDis \| 2 \| 142.94351 \| 103.85313 \| 0 \| 397.06297 \| 3.6963 \| 2.9508 \| \|  \| Total \| 19 \| 3867 \| 990.08305 \| 1445 \| 6290 \| 100.0000 \|  \| \| **Frequency Missing = 8** \| \| \| \| \| \| \| \| \| |
| 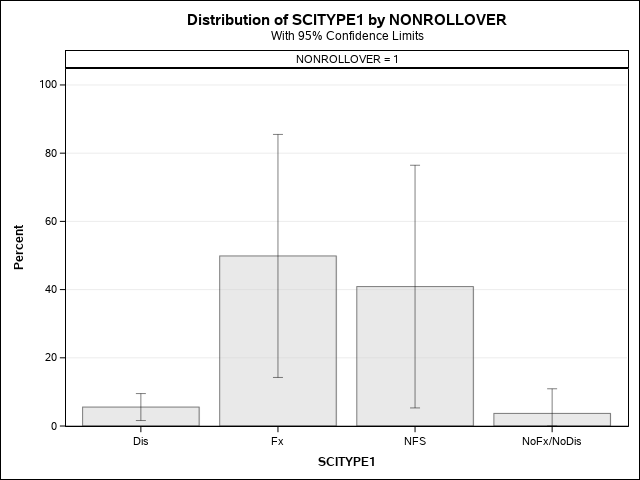 |

Table S2: SAS output for CISS (2017–2022) sub-analysis filtering for non-ejected occupants whose injuries likely occurred inside the rolling vehicle*.*

| \| Data Summary \| \| \| --- \| --- \| \| Number of Strata \| 12 \| \| Number of Clusters \| 32 \| \| Number of Observations \| 12196 \| \| Sum of Weights \| 7068086.94 \| |
| --- | --- | --- | --- | --- | --- | --- | --- | --- | --- | --- |
|  |
| \| Table of ROLLOVERCAUSED \| \| \| \| \| \| \| \| \| --- \| --- \| --- \| --- \| --- \| --- \| --- \| --- \| \| ROLLOVERCAUSED \| Frequency \| Weighted Frequency \| Std Err of Wgt Freq \| 95% Confidence Limits for Wgt Freq \| \| Percent \| Std Err of Percent \| \| 1 \| 9 \| 859.64447 \| 417.25256 \| 0 \| 1733 \| 2.0466 \| 0.9463 \| \| 2 \| 241 \| 41144 \| 5364 \| 29917 \| 52372 \| 97.9534 \| 0.9463 \| \| Total \| 250 \| 42004 \| 5477 \| 30540 \| 53468 \| 100.0000 \|  \| \| Frequency Missing = 11946 \| \| \| \| \| \| \| \| |

| Odds Ratio and Relative Risks (Row1/Row2) | | | |
| --- | --- | --- | --- |
| Statistic | Estimate | 95% Confidence Limits | |
| Odds Ratio | 1.6555 | 0.5490 | 4.9922 |
| Column 1 Relative Risk | 1.6491 | 0.5528 | 4.9199 |
| Column 2 Relative Risk | 0.9962 | 0.9855 | 1.0069 |
| Sample Size = 12196 | | | |

Table S3: NASS-CDS (2010–2015, model years ≥ 2002) summary of the a) raw counts, b) weighted data for the three combined injury categories and all exposed occupants, and c) comparison of rollover proportions and relative risks between this and the two main analyses. Also shown are the 95th percentile confidence intervals (CI) for the weighted data/relative risks.

| a) Raw counts |  |  |  |  |  |  |
| --- | --- | --- | --- | --- | --- | --- |
| Injury Category | Rollover |  | Non-Rollover |  | Total |  |
| All-VI (VI + VCI) | 113 |  | 236 |  | 349 |  |
| All-CI (CI + VCI) | 13 |  | 50 |  | 63 |  |
| All-Injuries (VI + CI + VCI) | 123 |  | 280 |  | 403 |  |
| All Exposed Occupants | 1,238 |  | 8,994 |  | 10,232 |  |
|  |  |  |  |  |  |  |
| b) Weighted data |  |  |  |  |  |  |
| Injury Category | Rollover | (95th CI) | Non-Rollover | (95th CI) | Total | (95th CI) |
| All-VI (VI + VCI) | 7,101 | (5,594–8,607) | 26,393 | (12,301 − 40,486) | 33,494 | (19,728 − 47,260) |
| All-CI (CI + VCI) | 598 | (114–1,082) | 4,933 | (2,088 − 7,779) | 5,531 | (2,586–8,476) |
| All-Injuries (VI + CI + VCI) | 7,587 | (5,811–9,363) | 29,789 | (15,972 − 43,607) | 37,376 | (23,478 − 51,274) |
| All Exposed Occupants | 279,339 | (208,683 − 349,995) | 2,919,485 | (1,956,254–3,882,717) | 3,198,824 | (2,172,333–4,225,316) |

c) Comparison

|  | All-VI | | | | All-CI | | | | All-Injuries | | | |
| --- | --- | --- | --- | --- | --- | --- | --- | --- | --- | --- | --- | --- |
|  | (VI + VCI) | | | | (CI + VCI) | | | | (VI + CI + VCI) | | | |
|  | Rollover proportion (95% CI) | | Relative Risk  (95% CI) | | Rollover proportion (95% CI) | | Relative Risk  (95% CI) | | Rollover proportion (95% CI) | | Relative Risk  (95% CI) | |
| NASS 1985 | 28.80% | (20.1–37.4) | 5.16 | (3.44–7.74) | 22.70% | (10.2–35.2) | 3.75 | (1.82–7.74) | 28.10% | (20.4–35.9) | 5.00 | (3.46–7.23) |
| NASS 2002 | 21.20% | (11.8–30.6) | 2.81 | (1.48–5.34) | 10.81% | (2.89–18.72) | 1.26 | (0.48–3.35) | 20.30% | (12.7–27.9) | 2.66 | (1.55–4.57) |
| CISS 2010 | 27.45% | (7.07–47.84) | 6.55 | (2.18–19.71) | 14.94% | (0.00–31.71) | 3.04 | (0.64–14.41) | 26.87% | (7.48–46.26) | 6.36 | (2.21–18.33) |
